# Marine macro-litter mass outweighs biomass in trawl catches along abyssal seafloors of Sardinia channel (Italy)

**DOI:** 10.1007/s11356-024-33909-3

**Published:** 2024-06-18

**Authors:** Ester Carreras-Colom, Maria Cristina Follesa, Laura Carugati, Antonello Mulas, Andrea Bellodi, Alessandro Cau

**Affiliations:** 1 Departament de Biologia Animal, Biologia Vegetal I Ecologia, Universitat Autònoma de Barcelona, Cerdanyola del Vallès, 08193 Barcelona, Spain; 2 Department of Life and Environmental Sciences, University of Cagliari, Via Tommaso Fiorelli 1, 09126 Cagliari, Italy; 3 ConISMa, ULR Cagliari, Consorzio Interuniversitario per le Scienze del Mare, Roma, Italy

**Keywords:** Marine litter, Monitoring, Deep-sea, Mediterranean Sea, Plastic pollution

## Abstract

This study provides new insights onto spatial and temporal trends of seafloor macro-litter in the abyssal seafloor of Sardinian channel, in central western Mediterranean (Italy). Trawl surveys were conducted at depths between 884 and 1528 m, thus focusing on one of the least investigated marine environments. None of the considered sites was litter free, with plastics being numerically dominant (57% of items), followed by metal (11%) and glass (16%). Recorded densities and weight ranged between 49.9 and 499 items km^−2^ and 1.4 and 1052 kg km^−2^. In the most contaminated sites, the weight of the litter collected in nets represented up to nine times the biomass of benthic megafauna, and, overall, in 60% of hauls macro-litter mass outweighed the biomass collected. Moreover, we report that megafauna was observed to be more abundant in sites where macro-litter presence was more severe. More studies are needed to elucidate the nature of this correlation, with biota being more abundant in hotspots of accumulation of seafloor macro-litter.

**Supplementary Information:**

The online version contains supplementary material available at 10.1007/s11356-024-33909-3.

## Introduction

The deep sea (i.e., below 200 m) represents the largest biome on Earth; yet, it is one of the least investigated (Danovaro et al. 2014). Despite being considered a fairly oligotrophic environment that has no primary production and relies on inputs from the surface, the limited information available indicates that the deep sea supports one of the highest levels of biodiversity on Earth, with unique and diverse communities of specialized organisms (Danovaro et al. 2017; WWF, IUCN 2004) and essential habitats for important commercial species (Cartes et al 2018; Cau et al. 2020). Albeit being at a long distance from the coast and urban settlements, the deep sea is not exempt from anthropogenic pressures, including waste dumping and littering (Fanelli et al. 2021; Levin and Le Bris 2015). During the last decades, there has been an increasing concern regarding marine litter, as evidence of its presence in the oceans worldwide has kept growing (Canals et al. 2021; Haarr et al. 2022). Marine macrolitter is hereby considered as the fraction of persistent, solid, human-related materials dispersed in the marine environment, that can be monitored by visual census and collected in bottom trawls. It has been reported from the deepest portions of the oceans to the most remote areas of the world, i.e., Antarctica (da Silva et al. 2023) or the Mariana Trench (Chiba et al. 2018). With estimates of up to 15 million tonnes of plastics alone entering the ocean each year (Forrest et al 2019), marine litter is likely to increase with time (Borrelle et al. 2020). Moreover, the interaction of marine litter with a wide array of organisms ranging from zooplankton to marine mammals (Jâms et al. 2020) has received great attention both in the scientific and popular media fields (MacLeod et al. 2021). Even though, to date, the problems associated with this waste are yet to be fully understood, the persistence of plastic waste in the environment should stir a continuous assessment to inform policies and address efficient mitigation actions, where relevant (Borrelle et al. 2020; Maximenko et al. 2019).

**Fig. 1:**
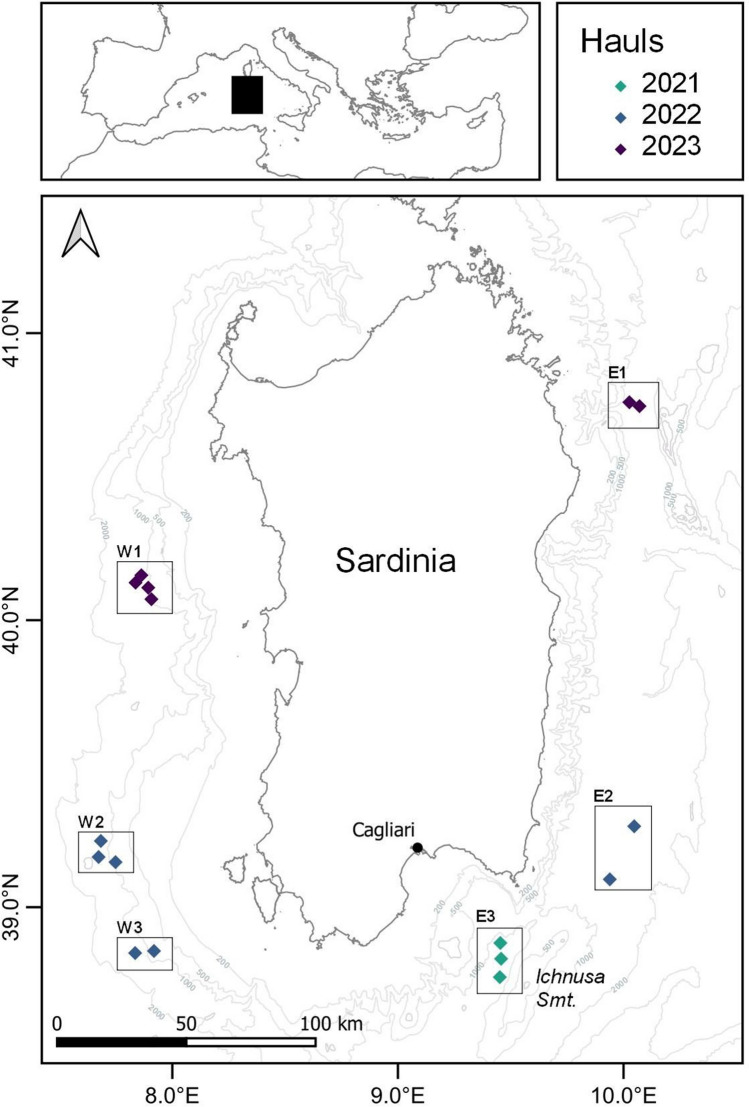
Study area and hauls carried out in 2021, 2022, and 2023. The location of key elements of the study area are indicated, i.e., the city of Cagliari (black dot) and the Ichnusa Seamount. For interpretation of the references to color in this figure legend, the reader is referred to the web version of this article

The Mediterranean is, comparatively, one of the most extensively surveyed areas on Earth. Besides foundational studies on the topic (e.g., Galgani et al. 2000), many opportunistic studies have been conducted throughout the Mediterranean basin (Alomar et al. 2020; Angiolillo et al. 2015; Ramirez-Llodra et al. 2011; Spedicato et al. 2019; Strafella et al. 2015; Tubau et al. 2015) which is widely acknowledged as one of the most polluted locations around the globe. The Mediterranean retains 5 to 10% of the global plastic mass dispersed on its surface (Suaria et al. 2016; Van Sebille et al. 2015), and heavily contaminated sites are reported on its bottom (Pierdomenico et al. 2019a). Despite these premises, it is still complicated to point out clear spatial or temporal trends; and this is particularly true for great depths (> 500 m) (Galgani et al. 2021). The deep-seafloor is, at the same time, the least studied environment due to the paucity of possible opportunistic surveys on marine litter but, at the same time, it is a pivotal environment to be studied since it constitutes the ultimate potential sink for the largest fraction of macro-litter, with plastic ahead (Haarr et al. 2022; Peng et al. 2020; Soto-Navarro et al. 2020; Woodall et al. 2014). In several areas of the Mediterranean Sea, the presence of a narrow continental shelf allows for the study of great depths thanks to their proximity to the coast, thus posing an excellent opportunity to cover this knowledge gap without the logistical constraints that deep surveys often entail. This study aims to extend the former assessment of marine macro-litter accumulation in the deep Mediterranean Sea by analyzing the density, weight, and composition of benthic marine litter through trawl surveys along the Sardinian continental slope and bathyal plain (Table 1; Fig. 1). The selection of this location offers an excellent opportunity to establish much-needed baseline values on the seafloor within the abyssal range. Methods of marine litter estimation followed those previously established to obtain readily comparable data. The influence of depth and location on marine litter abundance and composition was analyzed to test as to whether high accumulation of litter occurs in deeper areas and that certain locations may be hotspots for litter accumulation. Moreover, the density of marine litter was compared to that of benthic megafauna (i.e., demersal fish, mollusks, and crustaceans), to test whether the anthropogenic mass collected at great depths could outweigh that of deep-sea *biota*.

**Table 1:** Data on the sampling fishing hauls performed including the geographical coordinates (decimal), swept surface (km^2^), and benthic litter densities in number and biomass (number of items and kilos km^−2^, respectively)

Haul ID	Year	Depth (m)	Haul duration (hour:minute)	Latitude (North)	Longitude (East)	Swept area (km^2^)	Litter density (n. items km^−2^)	Litter density (kg km^−2^)
1PSP21*	2021	884	1:20	37.916	9.062	0.1393	0.0*	0.0*
2PSP21*	2021	1050	1:46	39.161	7.736	0.1874	0.0*	0.0*
3PSP21	2021	1050	1:49	38.829	9.444	0.2066	72.7	20.3
4PSP21	2021	1207	1:23	39.234	7.669	0.1197	74.9	13.8
5PSP21	2021	1017	1:17	38.830	9.460	0.1092	499	1052
1PSP22	2022	1050	1:00	39.178	7.660	0.1008	130	1.4
2PSP22	2022	1115	1:45	38.766	9.454	0.2939	61.5	47.6
3PSP22	2022	1228	1:30	38.845	7.829	0.2172	86.3	2.4
4PSP22	2022	970	1:49	38.885	9.457	0.2436	204	227
5PSP22	2022	927	1:00	38.853	7.913	0.1218	123	20.6
6PSP22*	2022	1418	2:00	39.115	9.984	0.2039	0.0*	0.0*
7PSP22	2022	1504	1:40	39.288	10.060	0.1802	49.9	6.3
8PSP22	2022	1416	1:34	39.104	9.948	0.1291	496	115
1PSP23	2023	1013	1:00	40.079	7.882	0.101	256	5.7
2PSP23	2023	994	1:55	40.119	7.867	0.177	299	9.2
3PSP23	2023	1380	1:22	40.136	7.809	0.133	45.0	0.5
4PSP23	2023	1205	1:50	40.162	7.835	0.163	97.9	126
5PSP23	2023	1169	1:15	40.766	10.061	0.120	91.9	11.0
6PSP23	2023	1310	0:46	40.752	10.107	0.126	63.5	2.2

^*^Considered non-valid because of evidence of damage on the fishing net or the lack of evidence that the apparatus reached the seafloor

## Materials and methods

### Sample collection

Three surveys were conducted in September 2021, November 2022, and September 2023, respectively. All hauls were carried out using a commercial fishing net (otter trawl bottom system) with a square mesh codend of 40 mm, on board a fishing vessel operating at depths > 850 m. All investigated sites were located at a distance comprised between 23 and 53 km from the nearest coastline (Fig. 1) and the specific locations were chosen based on previous surveys (i.e., S and SE of Sardinia; Cau et al. 2018) as well as for the condition of unexplored locations (W and NE). The swept surface (in km^2^) of each haul was estimated by multiplying the horizontal net opening by the distance trawled for each haul (obtained from GPS marks). The horizontal net opening was estimated from observed values under similar conditions (vessel speed and depth range) using the SCANMAR acoustic system in previous surveys (Cau et al. 2018).

### Sample analysis

Litter and megafauna quantification was carried out following the standardized protocol from the Mediterranean International Trawl Survey (MEDITS) handbook. Briefly, litter items collected from each haul were counted, weighted (± 0.1 g), and divided into 9 major categories and 27 subcategories (MEDITS Working Group 2017). Major categories included the following: plastic, rubber, metal, glass, paper, clothes/textile, wood, others and unspecified, while relative sub-categories are listed in Supplementary Table 1. The densities of marine litter in terms of no. of items per km^−2^ and kg km^−2^ for each major category were estimated by dividing the total abundance and weight of marine litter by the swept area of each haul. The swept area was estimated using the distance trawled for each haul and horizontal opening of the net (see details in Alvito et al. 2018). Similarly, the abundance and biomass of marine megafauna, consisting of the larger body-sized organisms associated with the seafloor (i.e., demersal fish and large metazoan invertebrate taxa), were estimated by counting and weighting all individuals of Elasmobranchs, Teleosts, Mollusks, and Crustaceans collected from each haul. The numerical and biomass density values were then obtained by dividing the total abundance and biomass by the swept area for each haul, thus providing the standardized values no. of organisms · km^−2^ and kg · km^−2^. In case of damaged and/or not identifiable organisms or biological by-catch, only the weight of identifiable remains of the former categories were considered, to be added for the estimation of biomass per km^−2^.

### Data processing

Heterogeneity of marine litter at each location was estimated through the Shannon–Wiener’s diversity index (H′), and Pielou’s evenness index (J′), where each marine litter category was considered as a “species” (Battisti et al. 2018). To test as to whether marine litter densities, in count and weight, differed with location (6 fixed levels; discriminated according to their geographic location regarding the island—see Fig. 1), latitude, longitude, or depth (range 884–1528 m), we used univariate analyses of variance (ANOVA) and simple linear regression models (LM). Univariate analyses were conducted for the total abundance of marine litter as well as for the most prevalent litter components (i.e., those representing > 20% of the total). Non-parametric analysis of variance (Kruskal–Wallis) and generalized linear models (GLM) were used when response variables (i.e., marine litter density values) did not follow a normal distribution even with a square-root transformation. GLM were fitted adapting the following general equation: *density values* ~ *geographic location* + *depth*, using the total count or weight density values of marine litter or that of a representative subcategory, and testing for each combination of depth with geographic-related variables, i.e., latitude, longitude, and the categorized geographic location. Similarly, uni- and multivariate analysis of variance (ANOVA and PERMANOVA) were used to test for differences among locations in the heterogeneity and composition of marine litter, respectively. PERMANOVA analyses were performed on Bray–Curtis matrices derived from square-root-transformed data.

A Mann–Whitney test was used to compare the mean densities of marine litter and megafauna collected for the whole bathymetric range and GLM were used to explore their correlation adding depth as a covariable. Briefly, these GLM were fitted using the density values of biomass count and biomass weight as response variables with marine litter density values (count and weight) and depth as predictor variables (i.e., *marine megafauna density* ~ *marine litter density* + *depth*). Finally, published data from the same location obtained during the period 2015–2017, thus representing an 8-year timespan (Cau et al. 2018), was also obtained and used to elaborate a temporal comparison on the evolution of marine litter distribution and characteristics whose significance was tested using a LM on square-root-transformed count density values.

## Results

**Fig. 2:**
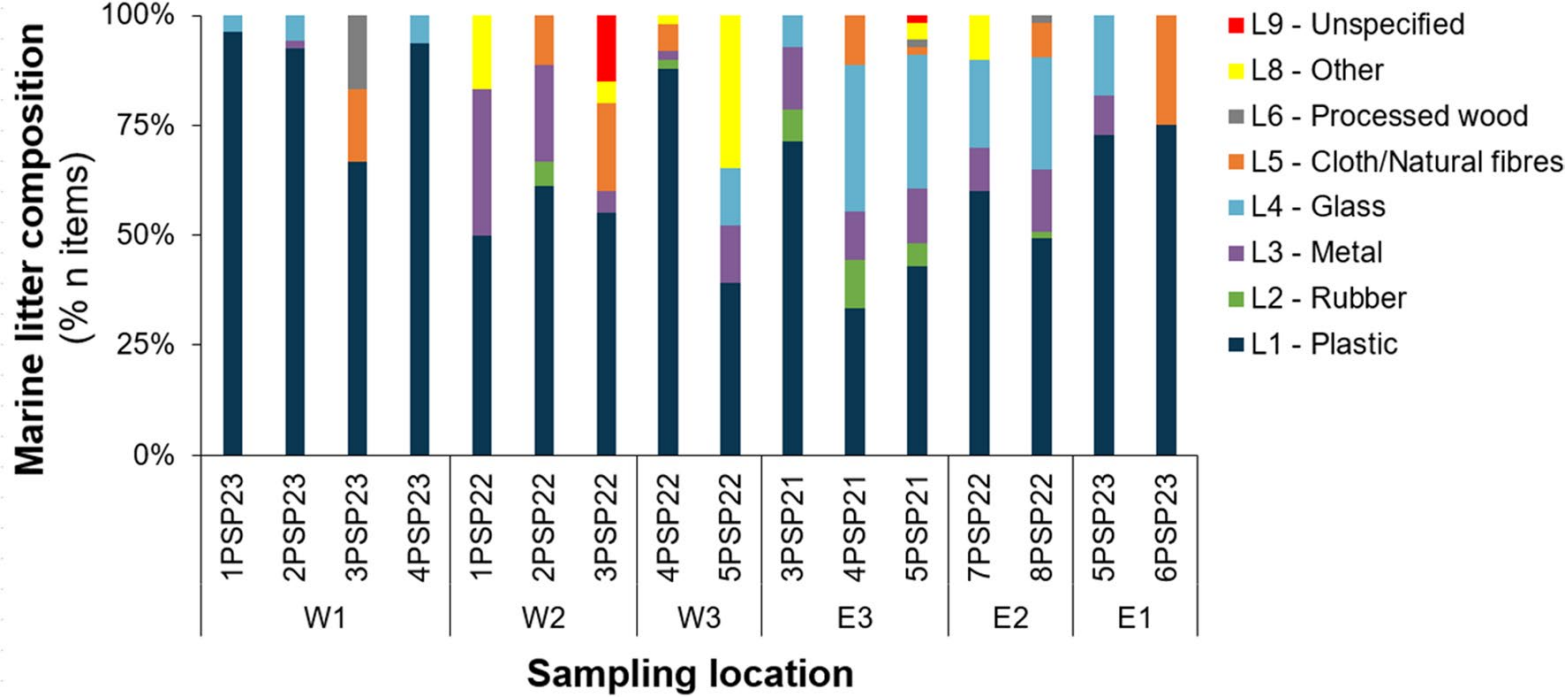
Relative composition of marine litter (in % from the total number of items collected) collected in this study according to the macro-categories of the MEDITS protocol and ordered in relation to their position. For more details on the geographic positions, see Table 1 and Fig. 1

A total of 19 hauls were performed over the 3-year period of which 16 were considered acceptable (i.e., no damage to the fishing net and evidence that the apparatus reached and worked on the seafloor). From these, 390 items with a total weight > 230 kg were collected. The total swept area was estimated to be 2.54 km^2^ and the depth range covered was from 884 to 1528 m. Macro-litter items were collected in all valid hauls with densities ranging between 45.0 and 509 items km^−2^ (average values of 167 ± 150) and 0.5 and 1052 kg km^−2^ (104 ± 261 on average) (Table 1). Plastic items were consistently dominant in almost all hauls and represented 66.8% of all items found (*n* = 258), followed by glass (L4; 12.7% of the total, *n* = 49), metal (L3; 8.5%, *n* = 33), clothes (L5; 4.9%, *n* = 19), and other items (L8; 3.6%, *n* = 14) (Fig. 2). In terms of weight, the composition was much more heterogeneous, with the category “other” contributing the most to the total values of marine litter (L8 34.9, 81 kg in total) and another five categories, including in decreasing order glass, metal, unspecified, rubber, and plastic, with % contributions ranging between 9.5 and 17.4%.

In four out of the six hauls where the category “other” was reported, the said elements consisted of tar agglomerates of varying sizes. Single-use plastic and metallic items (e.g., plastic bags, food packaging, and drinking cans) were the most common items found (67.4 and 8.9%, respectively), and among the glass items, 87.8% were bottles (Supplementary Material, Table S1). Fishing-related items accounted for < 6% of the litter both in terms of abundance and weight. More specifically, the most collected items were, in this order of abundance, plastic bags (*n* = 110), food packaging containers of varied colors and sizes (*n* = 58), glass bottles (*n* = 43), and metallic drinking cans (*n* = 20).

**Fig. 3:**
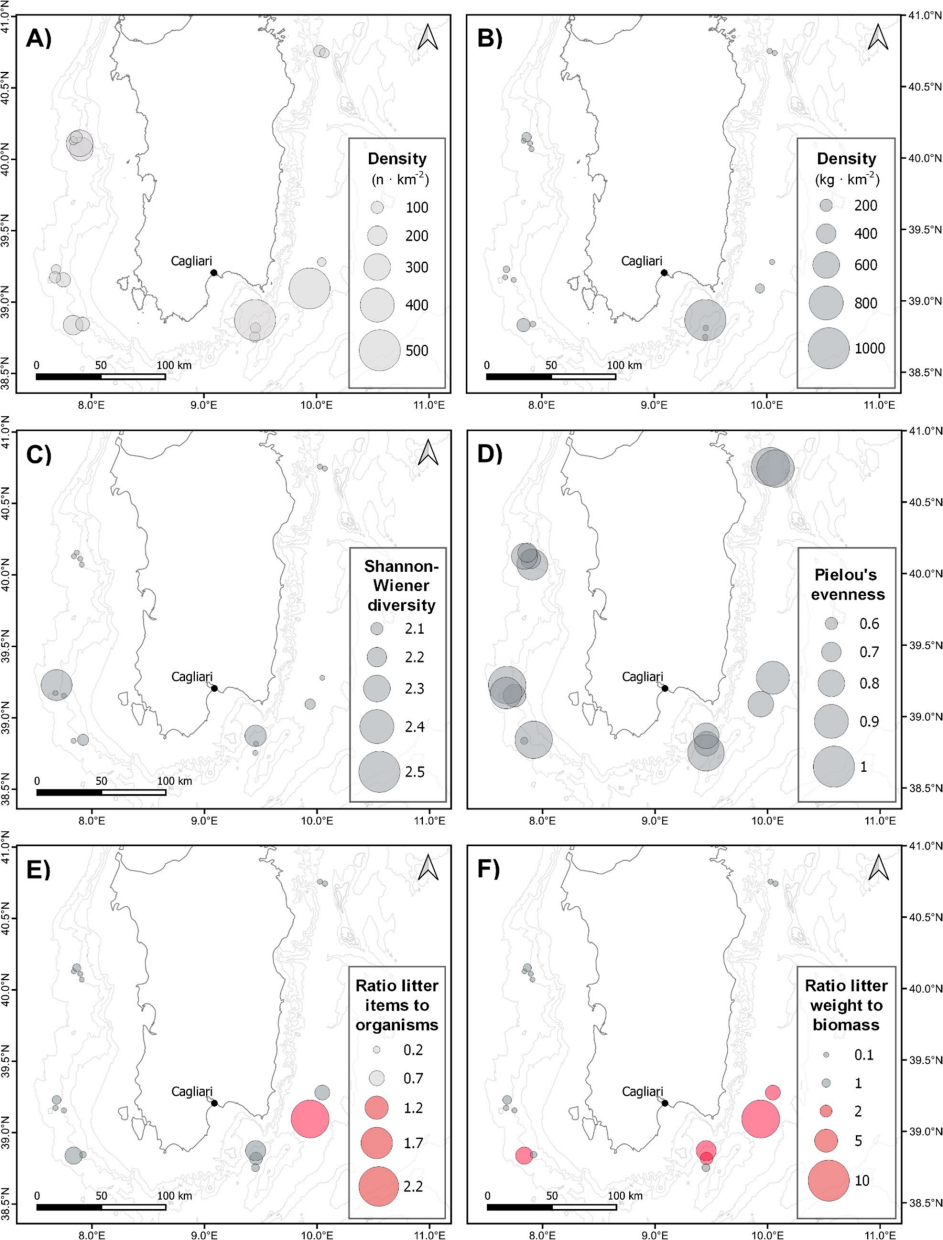
Geographical distribution of total marine litter densities (*n* items km^−2^ and kg km.^−2^), diversity indices for marine litter composition (Shannon–Wiener and Pielou’s evenness), and ratios of marine litter to megafauna densities (numerical and weight). The red color represents hauls where marine litter abundance (*n*) or weight surpassed that of megafauna (i.e., ratio of marine litter items to megafauna organisms > 1

Uni- and multivariate analyses revealed almost no significant trends in marine litter density, neither for total nor for each of the most prevalent subcategories (i.e., plastic, glass, metal and other), with sampling location or longitude. The total marine litter count and weight densities and plastic count density were negatively correlated with depth (GLM; *z* =  − 10.71, *p* > 0.001, *z* =  − 26.456, *p* < 0.0001, and *z* =  − 12.95, *p* < 0.001, respectively, Table 2). Latitude was a significant factor only for the density of metallic items, with higher densities at the lowest latitudes (LM; *t* =  − 2.732, *p* = 0.0162). Shannon–Wiener diversity index (H′) indicated that the most diverse litter items were found on 5PSP21 and 2PSP22 with values of 2.4 and 2.2, respectively, while the least diverse was 3PSP23 (H′ = 0.87). At the same time, the Pielou’s evenness index (J′), with values ranging from 0.7 to 0.95 indicated that the subcategories of litter items were mostly evenly distributed, except for 4PSP22, which exhibited a lower evenness (J′ = 0.53). No significant trends were observed for marine litter heterogeneity indices with any of the spatiotemporal factors tested nor with marine litter descriptors (count and weight densities; ANOVA, *p* > 0.05). Representations of the geographical distribution of the main descriptors for marine litter revealed a scattered disposition of values of each descriptor (Fig. 3). The PERMANOVA tests showed the absence of any significant differences in the seafloor macrolitter composition among locations.

**Table 2:** Summary of relevant results from generalized linear models (GLM) applied to marine litter abundance and marine biomass (count and weight) to test the contribution of depth the along abyssal seafloors of Sardinia channel. *** *p* < 0.001 and *n.s* not significant (*p* > 0.05)

Parametric coefficients:	Estimate	Std. error	*z* value	Pr( <|*z*|)
Marine litter count				
(Intercept)	6.7599	0.1525	44.32	***
Depth	− 0.0014	0.0001	− 10.71	***
Marine litter weight				
(Intercept)	11.4958	0.2494	46.10	***
Depth	− 0.0061	0.0002	− 26.46	***
Biomass count				
(Intercept)	8.688	0.096	90.50	***
Marine litter count	0.0025	0.00006	37.55	***
Depth	− 0.0026	0.00008	− 31.78	***
Biomass weight				
(Intercept)	8.700	0.1054	82.53	***
Marine litter count	0.0012	0.00003	43.24	***
Depth	− 0.0023	0.00009	− 25.61	***
Biomass count				
(Intercept)	7.266	0.3050	23.82	***
Marine litter weight	0.0026	0.0002	13.01	***
Depth	− 0.00335	0.0003	− 12.69	***
Biomass weight				
(Intercept)	7.2033	0.3337	21.59	***
Marine litter weight	0.0013	0.00008	16.60	***
Depth	− 0.0030	0.0003	− 10.28	***

**Fig. 4:**
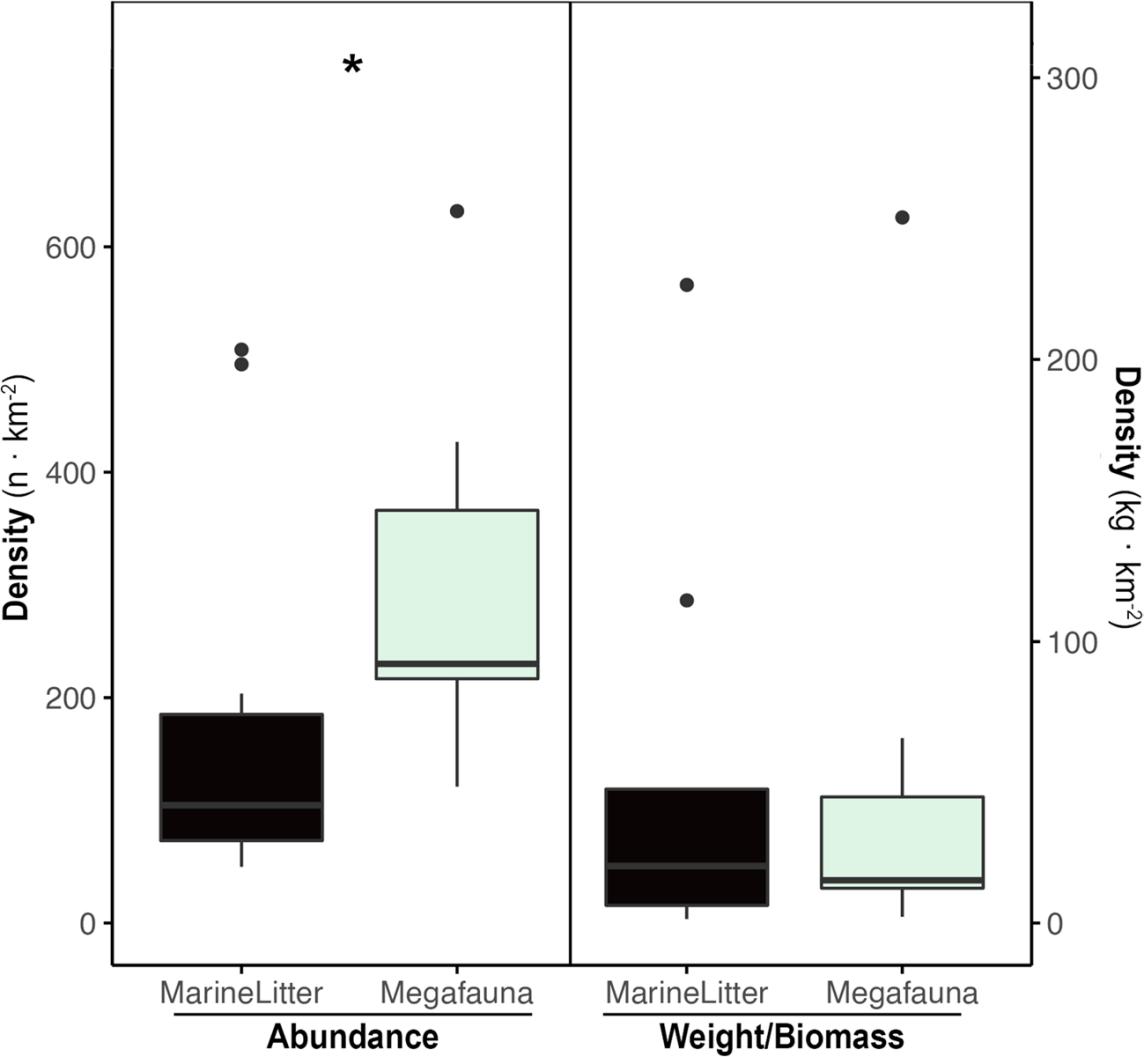
Mean values of abundance and weight km^−2^ (± std. error) of marine litter and marine megafauna. To improve visualization, two data points with 1998 *n* megafauna km^−2^ and 1052 kg marine litter km^−2^ were removed from the abundance and weight/biomass plots, respectively. Significant differences (Mann–Whitney-Wilcoxon test for comparison; *p* < 0.05) are presented with *

**Fig. 5:**
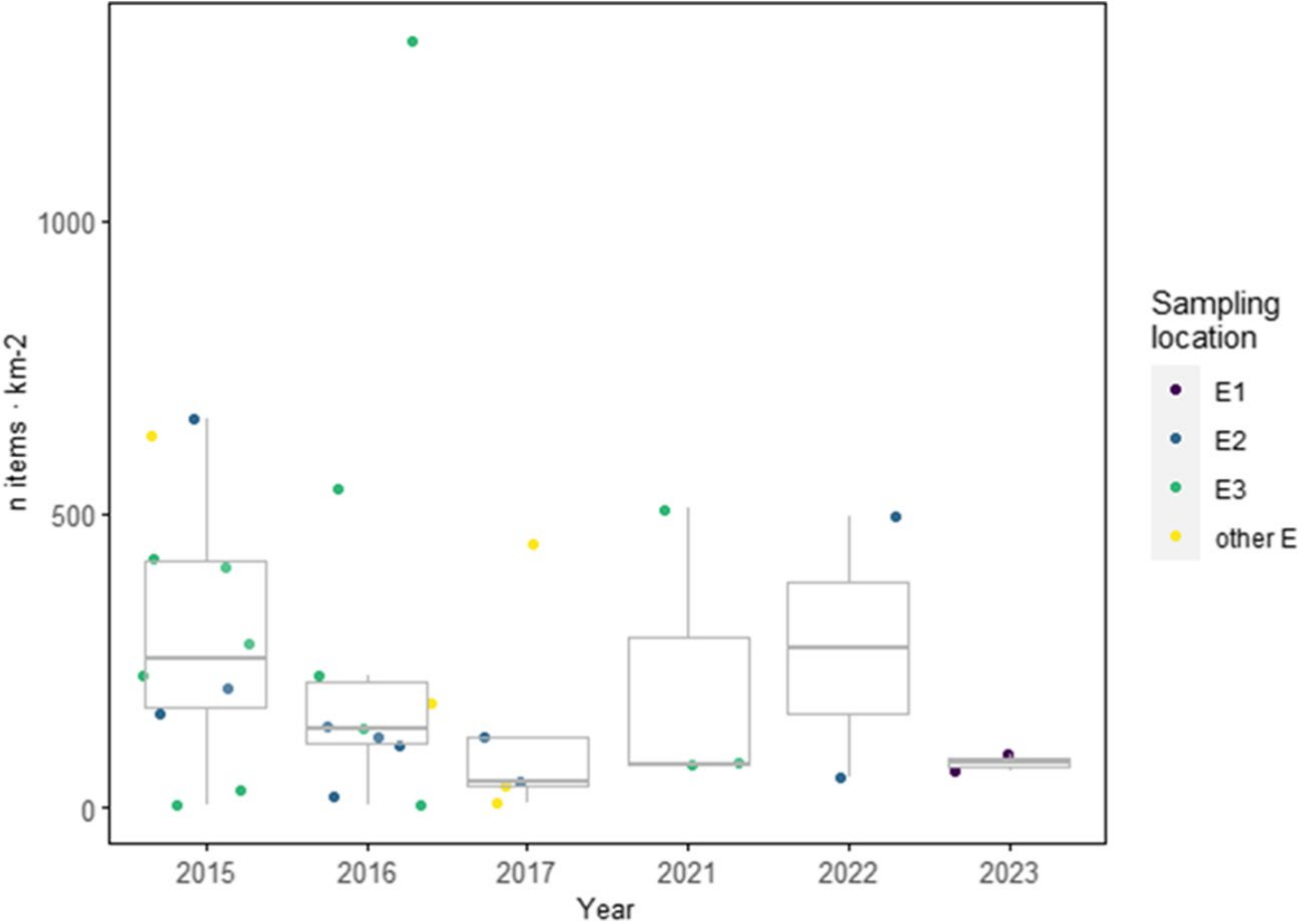
Marine litter density (*n* items km.^−2^) observed during the period 2015–2017 (Cau et al. 2018) and 2021–2023 (this study) for the eastern sampling locations of Sardinia (see details on codes for location in Table 1 and Fig. 1)

The megafauna density ranged between 121 and 1989 organisms and 2.2 and 250 kg km^−2^. Significant differences were observed in terms of numerical densities, with megafauna being much more abundant than litter (Mann–Whitney-Wilcoxon test; *p* = 0.00055), but with no differences found in terms of weight/biomass (*p* = 0.299) (Fig. 4). Both counts and weight of marine litter were positively associated with megafauna counts and biomass as well as negatively with depth (GLM; *p* < 0.05; see test statistics and *p*-values in Table 2). Finally, no significant trends with year were found for the number of items when comparing our results in the southeastern locations with available published data (LM; *p* < 0.05, Fig. 5).

## Discussions

Our results point to a higher dispersion of marine litter throughout the coast of Sardinia, reaching depths > 1000 m and with single-use plastic making up for the major fraction and thus following global trends (Haarr et al. 2022; Pham et al. 2014). The reported values fall within the range of those previously described in the area by Cau et al. (2018) during the period 2015–2017 with similarities in terms of the variability reported and relative dominance of macrocategories (i.e., plastic, glass, metal, and cloth). Overall, fishing-related items were not considered abundant and represented less than 6% of the overall items found, which is consistent with the low relative abundance reported for shallower areas in Sardinia (i.e., 9.4%; Alvito et al. 2018), but still contrasts with results reported from neighboring areas where relative abundance exceeded 20% (Garofalo et al. 2020). This might be due to the more intense fishing activities conducted in the depth range covered by the MEDITS trawl survey (up to 800 m), specifically focused on fishing grounds. Single-use items of either plastic or metal, mostly including bags, tableware, and food and drink containers, were the most frequent items encountered, suggesting either the influence of water currents in the transport from land-based sources (i.e., Flummini Mannu discharging into the Gulf of Cagliari; Alvito et al. 2018) or an important influence of shipping lanes. In any case, these results highlight that the main source of marine pollution may be improper waste disposal or even the direct illegal dumping of these items into the environment in domestic or recreational activities (Munari et al. 2016). To a lesser extent, the presence of concrete and metal objects, heavy litter that most likely originates from direct sea-based point sources, also suggests illegal dumping in highly trafficked areas (Alvito et al. 2018). Furthermore, it is worth noticing how trawls (especially commercial nets) can easily underestimate the amount of low-density and/or small items that could pass through the net mesh, and thus, results here could be an underestimation of the actual situation (Canals et al. 2021; Nogueira et al. 2023; Watters et al. 2010).

Another frequent finding in the Southern part of Sardinia were tar agglomerates, i.e., oil conglomerates that originate from marine oil pollution, which are a clear sign of oil spill, accidental loss, or reckless dumping from commercial shipping activities occurring in the proximity of the investigated sites. However, depending on the size of tar agglomerates, they could originate from floating agglomerates that, after a more or less long period, become fouled or weathered and thus sink to the seafloor (Warnock et al. 2015). In our study, we did not observe tar agglomerates associated with plastic items (i.e., “plastitar”), which occurrence has been recently documented (Saliu et al. 2023). The overall absence of significant spatial trends in the distribution, composition, and abundance of marine litter provides support once again to the great heterogeneity and scattering of marine litter on the seafloor (Woodall et al. 2015). Of all the marine litter components identified, only metal items showed a slight spatial trend, being more abundant on the southern side of the island, particularly on the SE locations. Moreover, despite not showing general trends when considering the whole area surveyed, glass, wood, and rubber items also appeared in higher abundance in the hotspots of litter identified in the SE area. These findings could be related to the narrow continental shelf on the eastern side of the island, deeply incised by the presence and activity of submarine canyons that might favor the transport of these high-density items, which would otherwise be expected to get stuck (e.g., buried) in the continental shelf (Canals et al. 2006; Pusceddu et al. 2013; Moccia et al. 2022, 2021). Overall, all subcategories of marine litter were rather evenly distributed across all hauls and exhibited similar diversity values, with few exceptions. The use of the diversity metrics (i.e., Shannon–Wiener’s diversity index and Pielou’s evenness index) for marine litter, although lacking the classical implications for ecological dynamics as in biological systems, may still provide useful information for these non-biological communities. As suggested by Battisti et al. (2018), these metrics might inform operational actions, serving as proxies for the complexity of the problem.

The three most contaminated locations represented almost half of all items collected (48.8%) and 70.9% of all the weight collected in the survey when density values were standardized by the swept area. These hauls were scattered (SE, S, and NW), thus hindering the establishment of one single accumulating area and once again supporting the idea that marine litter distribution on the seafloor is highly heterogeneous and driven by several factors, among which (i) intrinsic features of macro-litter items, which eventually influence their transport, sink, and persistence in the environment, combined with (ii) local circulation patterns (e.g., upwelling or downwelling); (iii) the presence of peculiar geomorphological features such as seamounts, submarine canyons, or shelf areas (Woodall et al. 2015; Pierdomenico et al. 2019a, b, 2023; Cau et al. 2022); and nonetheless, (iv) anthropogenic variables (Galli et al. 2023). On the other hand, the remaining hauls showed a much smaller density of marine litter (< 90 items and 20 kg km^−2^ on average), which would support Sardinia as being a less impacted area compared to other particular regions of the Mediterranean (i.e., NW Mediterranean Sea or Adriatic Sea) (Alvito et al. 2018; Galimany et al. 2019; Soto-Navarro et al. 2020).

Many knowledge gaps exist regarding the interaction between marine litter and benthic megafauna such as demersal fish, crustaceans, and mollusks (Deudero and Alomar 2015). However, as with any other environmental pollutant, the higher the abundance and availability in the environment the higher the risk it may entail. As a result of the dawned opinion on plastics and their rapid increase in the environment, the thought of plastic items surpassing fish at a certain point in time has spread. The comparison of standardized counts on marine litter items and megafauna revealed that, although megafauna still dominates in terms of counts, the weight of marine litter is equivalent to that of megafauna, with three of the hotspots for marine litter identified having between three and up to nine times more litter than biomass. Although single-use items (likely underestimated in this study) fairly represented 10% of the total weight of marine litter, it is obvious that we need a major change to tackle the pollution problem as a whole.

Something worth noting regarding the interaction between marine litter and megafauna is that a slight positive correlation was observed, likely confirming how in certain cases, within poorly structured environments, even macro-litter can become a source of habitat heterogeneity and somehow attract biodiversity, compared to surrounding environments (Carugati et al. 2021; Song et al. 2021). Besides this, which could be one but hardly be the only explanation, the observed pattern could likely mirror the major transport processes that drive both the transport of marine litter items as well as nutrients that support the megafaunal community (Tubau et al. 2015; Sanchez-Vidal et al. 2013). Moreover, this positive correlation was inversely correlated to depth, with both the abundance of megafauna and marine litter being slightly higher at the shallower range of depths studied. This decreasing trend in the abundance of megafauna, highly dominated by large fish at these depths, has been long known in the area (Follesa et al. 2011), yet when it comes to marine litter, it refutes the simplistic thought that all marine litter travels to deeper areas (Canals et al. 2021; Peng et al. 2020) or at least that this should be evidenced by a decreasing gradient. Although it might eventually be the case, our results prove that transport and distribution processes are much more complex and are likely to affect each of the compartments of marine litter differently, as well as be influenced by the geomorphological features of the area.

Despite evidence of increasing quantities of litter on the seafloor in certain locations (Gerigny et al. 2019; Tekman et al. 2017), other studies working with a similar time span have reported conflicting results when trying to define temporal trends (García-Rivera et al. 2018). Our limited time scale does not allow for a proper temporal analysis on its own; however, when analyzed together with previously reported data, it fails to point to any particular trend, with values in 2022 being similar to those observed throughout 2015 and 2016. The lower values observed in 2023 might respond to geographical differences rather than a temporal trend since it represented the northernmost surveyed area. Overall, this suggests that the standing stock in the surveyed area dwells in an equilibrium status between input and output of seafloor macro-litter. The “output” likely involving secondary dispersion mediated by human activities such as dredging and trawling, which is less likely to occur at such depths, but also the progressive burial due to sediment flows and resettling of sedimentary particles (Tubau et al. 2015). One location off the city of Cagliari at c.a. 1000 m depth consistently showed some of the highest densities each year. Such accumulation hotspot might be the result of the proximity to sources (i.e., Cagliari metropolitan area, riverine input, maritime traffic) working together with the transport processes and the peculiar local geomorphology (i.e., the presence of the Ichnusa Seamount and narrowness of the continental shelf) (García-Rivera et al. 2018; Woodall et al. 2015). The presence of natural debris, i.e., *Posidonia* aegagropilae, in addition to the mixture of land- and marine-sourced litter, evidences an efficient transport from the surface to the floor in the area (Pierdomenico et al. 2019a; Tubau et al. 2015). Similarly, in the NW Mediterranean Sea, the narrowing of the continental shelf coupled with the presence of submarine canyons has been pointed out as key elements to favor trapping and funneling of sediments and debris to the abyssal plains (Canals et al. 2006; Tubau et al. 2015). The last survey highlighted how, although to a lesser extent, the western coast also represented an area of greater accumulation than the northeastern area. Such accumulation patterns might respond again to a variety of factors of which, as pointed out already by Cau et al. (2022), including the south-eastward current coming from the Balearic Islands, the Western Sardinian Current, might play a key role favoring the transport of low-density materials like plastics.

Overall, our findings corroborate how the Sardinia channel could represent a major accumulation hotspot for macro-litter. The proximity and overlap between organism distribution and macro-litter accumulation hotspots might enhance the chances of direct or indirect interaction. Accidental ingestion of anthropogenic particles (particularly microplastics) is just the most widely documented interaction and encompasses a wide range of fauna, from small crustaceans to top predators (e.g., Franceschini et al. 2021; Sbrana et al. 2022), but is not the only. Other examples are entanglement of animals, which may have their ability to catch food or avoid predators impaired, or incur wounds from mechanical abrasion or cutting action litter (Browne et al. 2015). On top of this, increasing evidence of how macro-litter accumulated in the seafloor could become a suitable substrate for encrusting, settling organisms, including alien species (Carugati et al. 2021; Song et al. 2021), deposition surfaces (Valderrama-Herrera et al. 2023), or even provide shelter to invertebrates and fish (Pierdomenico et al. 2019b). In broad terms, the presence of marine litter may contribute to higher levels of spatial heterogeneity, which have been pointed out as driving factors for the faunal assemblages in deep-sea areas (Pierdomenico et al. 2019b). Hence, the identification of such areas should lead to future efforts to better understand the consequences of these interactions, which eventually render accumulation areas as potential biodiversity hotspots. This is particularly relevant for deep-sea environments, where likely only slow environmental processes influence the standing stock of litter, rather than anthropogenic (i.e., commercial trawlers) as it usually happens at shallower depths (Franceschini et al. 2019), rendering these accumulation hotspots more stable in time and likely to have more interactions with fauna.

Besides this, the observed dominance of plastic, together with other single-use items among the marine litter analyzed, highlights the type of pollution that affects the deeper waters in the western and south of Sardinia hence pointing out how a major lifestyle change is needed to tackle the problem at its source. Therefore, political and management efforts should prioritize the prevention or mitigation of illegal dumping, either in recreational activities, or in highly trafficked areas. The present work provides a further assessment of the distribution of marine litter around the deep waters of Sardinia, thus stepping one step closer to understanding the spatial heterogeneity in the area, much needed to potentially address remediating actions (Haarr et al. 2022). Overall, our findings reveal a heterogeneous distribution of marine litter on the seafloor around Sardinia, with hotspots of accumulation driven by the combination of local geomorphological and hydrodynamic factors. Most importantly, the prevalence of single-use plastics and the interaction between marine litter and megafauna underscore the urgent need for effective waste management and pollution mitigation strategies.

### Supplementary Information

Below is the link to the electronic supplementary material.

Supplementary file1 (DOCX 36 KB)

## Data Availability

Data will be made available from the authors upon reasonable request.
