# Supplementary material for: Marine macro-litter mass outweighs biomass in trawl catches along abyssal seafloors of Sardinia channel (Italy)
Source: Environ Sci Pollut Res Int. 2024 Jun 18;31(30):43405–16. doi: 10.1007/s11356-024-33909-3 (PMC11222263; doi:10.1007/s11356-024-33909-3)
Supplement: Supplementary file 1 — Supplementary file1 (DOCX 36 KB) [file 11356_2024_33909_MOESM1_ESM.docx]

Marine macro-litter mass outweighs biomass in trawl catches along abyssal seafloors of Sardinia channel (Italy)

Ester Carreras-Colom^1,2^, Maria Cristina Follesa^2,3^, Laura Carugati^2,3^, Antonello Mulas^2,3^, Andrea Bellodi^2,3^ & Alessandro Cau^2,3🖂^

^1^Departament de Biologia Animal, Biologia Vegetal i Ecologia, Universitat Autònoma de Barcelona, Cerdanyola del Vallès, 08193 Barcelona, Spain

^2^Università degli Studi di Cagliari, Dipartimento di Scienze della Vita e dell’Ambiente, Via Tommaso Fiorelli 1, 09126 Cagliari, Italy

^3^ Consorzio Interuniversitario per le Scienze del Mare, CoNISMa, ULR Cagliari, Cagliari, Italy

🖂**Corresponding Authors:**

Telephone: +39 070 675 6626;

E-mail: [alessandrocau@unica.it](mailto:alessandrocau@unica.it);

**Number of pages: 3**

**Number of tables: 2**

**Table S1**. Composition of marine litter collected in this study according to the nine major categories (L1 to L9) of the MEDITS protocol, with corresponding subcategories. *n* = number of items.

| **Category and subcategory** | **n** | **% over category** | **% over total** | **Weight (g)** | **% over category** | **% over total** |
| --- | --- | --- | --- | --- | --- | --- |
| **L1 - Plastic** | **260** |  | **66.7** | **22188** |  | **10.7** |
| Bags | 110 | 42.3 | 28.2 | 5385 | 24.3 | 2.6 |
| Bottles | 8 | 3.1 | 2.1 | 460 | 2.1 | 0.2 |
| Food packaging | 58 | 22.3 | 14.9 | 737 | 3.3 | 0.4 |
| Sheets | 2 | 0.8 | 0.5 | 390 | 1.8 | 0.2 |
| Hard plastic objects | 15 | 5.8 | 3.8 | 660 | 3.0 | 0.3 |
| Fishing nets | 5 | 1.9 | 1.3 | 4255 | 19.2 | 2.0 |
| Fishing lines | 9 | 3.5 | 2.3 | 3195 | 14.4 | 1.5 |
| Other fishing | 5 | 1.9 | 1.3 | 373 | 1.7 | 0.2 |
| Synthetic ropes | 12 | 4.6 | 3.1 | 1233 | 5.6 | 0.6 |
| Others | 36 | 13.8 | 9.2 | 5500 | 24.8 | 2.6 |
| **L2 - Rubber** | **8** |  | **2.1** | **23830** |  | **11.4** |
| Tyres | 1 | 12.5 | 0.3 | 5000 | 21.0 | 2.4 |
| Other | 7 | 87.5 | 1.8 | 18830 | 79.0 | 9.0 |
| **L3 - Metal** | **33** |  | **8** | **36701** |  | **17.6** |
| Drinking cans | 20 | 60.6 | 5.1 | 1420 | 3.9 | 0.7 |
| Food cans | 4 | 12.1 | 1.0 | 135 | 0.4 | 0.1 |
| Containers | 2 | 6.1 | 0.5 | 4400 | 12.0 | 2.1 |
| Barrels | 2 | 6.1 | 0.5 | 29560 | 80.5 | 14.2 |
| Cables | 3 | 9.1 | 0.8 | 1105 | 3.0 | 0.5 |
| Fishing-related | 2 | 6.1 | 0.5 | 81 | 0.2 | 0.0 |
| War remnants | 0 | 0.0 | 0.0 | 0 | 0.0 | 0.0 |
| **L4 - Glass** | **49** |  | **13** | **40575** |  | **19.5** |
| Bottles | 43 | 87.8 | 11.0 | 14070 | 34.7 | 6.8 |
| Pieces of glass | 2 | 4.1 | 0.5 | 1785 | 4.4 | 0.9 |
| Ceramic | 1 | 2.0 | 0.3 | 20 | 0.0 | 0.0 |
| Large objects | 3 | 6.1 | 0.8 | 24700 | 60.9 | 11.9 |
| **L5 - Cloth/Natural fibres** | **19** |  | **4.9** | **2875** |  | **1.4** |
| Clothing | 5 | 26.3 | 1.3 | 830 | 28.9 | 0.4 |
| Large pieces | 6 | 31.6 | 1.5 | 1870 | 65.0 | 0.9 |
| Natural fibres | 8 | 42.1 | 2.1 | 175 | 6.1 | 0.1 |
| Hygienic/Sanitary | 0 | 0.0 | 0.0 | 0 | 0.0 | 0.0 |
| **L6 - Processed wood** | **3** |  | **0.8** | **360** |  | **0.2** |
| **L7 - Paper and cardboard** | **0** |  | **0.0** | **0** |  | **0.0** |
| **L8 - Other** | **14** |  | **3.6** | **81130** |  | **39.0** |
| **L9 - Unspecified** | **4** |  | **1.0** | **25005** |  | **12.0** |

**Table S2**. Density in number of organisms and weight per km^2^ of megafauna collected in this study according to the following categories: fish, crustaceans, sharks and cephalopods.

| **Haul ID** | **Density**  **(n organisms · km^-2^)** | | | | | **Density**  **(kg biomass · km^-2^)** | | | | |
| --- | --- | --- | --- | --- | --- | --- | --- | --- | --- | --- |
|  | Teleost fish | Sharks | Crustaceans | Cephalopods | Total | Teleost fish | Sharks | Crustaceans | Cephalopods | Total |
| 3PSP21 | 49 | 15 | 53 | 4.8 | 121 | 1.8 | 2.4 | 0.24 | 4.8 | 9.3 |
| 4PSP21 | 83 | 67 | 208 | 8.3 | 366 | 3.4 | 12 | 1.3 | 0.04 | 17 |
| 5PSP21 | 842 | 943 | 176 | 28 | 1989 | 84 | 164 | 0.97 | 1.3 | 250 |
| 1PSP22 | 90 | 50 | 90 | 0.0 | 230 | 7.4 | 6.0 | 0.38 | 0 | 14 |
| 2PSP22 | 96 | 157 | 55 | 0.0 | 307 | 11 | 32 | 0.36 | 0 | 43 |
| 3PSP22 | 55 | 64 | 95 | 9.1 | 223 | 1.7 | 10 | 0.38 | 0.07 | 12 |
| 4PSP22 | 196 | 346 | 86 | 4.1 | 632 | 25 | 41 | 0.45 | 0.20 | 66 |
| 5PSP22 | 115 | 189 | 115 | 8.2 | 427 | 8.5 | 36 | 0.55 | 0.25 | 45 |
| 7PSP22 | 28 | 5.5 | 133 | 0 | 166 | 0.33 | 1.4 | 0.53 | 0 | 2.2 |
| 8PSP22 | 39 | 7.7 | 155 | 16 | 217 | 9.8 | 1.5 | 0.75 | 0.43 | 13 |
| 1PSP23 | 187 | 296 | 109 | 49 | 641 | 22 | 57 | 0.24 | 1.3 | 81 |
| 2PSP23 | 350 | 367 | 124 | 17 | 859 | 18 | 62 | 0.84 | 0.28 | 81 |
| 3PSP23 | 45 | 30 | 68 | 15 | 158 | 2.8 | 2.6 | 0.36 | 0.20 | 5.9 |
| 4PSP23 | 312 | 300 | 43 | 31 | 685 | 37 | 112 | 0.39 | 0.63 | 150 |
| 5PSP23 | 359 | 484 | 175 | 0 | 1019 | 44 | 72 | 0.86 | 0 | 117 |
| 6PSP23 | 64 | 40 | 87 | 7.9 | 198 | 4.2 | 7.7 | 0.18 | 0.26 | 12 |
